# Health-Risk Factors and the Prevalence of Chronic Kidney Disease: Cross-Sectional Findings from a National Cohort of 87 143 Thai Open University Students

**DOI:** 10.5539/gjhs.v7n5p59

**Published:** 2015-02-24

**Authors:** Prasutr Thawornchaisit, Ferdinandus de Looze, Christopher M Reid, Sam-ang Seubsman, Thanh Tam Tran, Adrian Sleigh, Thai Cohort Study Team

**Affiliations:** 1 Lerdsin General Hospital, Bangkok, Thailand; 2 School of Medicine, University of Queensland, Australia; 3 School of Public Health and Preventive Medicine, Monash University, Australia; 4 School of Human Ecology, Sukhothai Thammathirat Open University, Thailand; 5 National Centre for Epidemiology and Population Health, ANU College of Medicine, Biology and Environment, The Australian National University, Australia

**Keywords:** CKD, demography, SES, BMI, diabetes, high lipids, hypertension, smoking, drinking, Thailand

## Abstract

**BACKGROUND:**

Chronic kidney disease (CKD) is becoming a major health challenge worldwide as its aetiology has transferred from predominantly infectious disease to emerging chronic diseases, especially diabetes and hypertension. A rapid health-risk transition driven by economic development is transforming Thailand which is now becoming an ageing country where chronic diseases are a major health burden.

**METHODS:**

This study used the 2005 baseline cross-sectional dataset of 87 143 Thai Cohort Study members to investigate risk factors associated with CKD. Using multivariate logistic regression, we looked into the relationship between CKD and demographic and socioeconomic factors, personal health status and various health-related behaviours.

**RESULTS:**

The prevalence of CKD in men was lower than that in women (2.5% vs 2.7%). In both sexes, CKD is associated with ageing, cigarette smoking and drinking alcohol, having diabetes, high lipids and hypertension. In men, CKD was associated with living in rural areas, having a low income, a higher BMI, short sleeping and having Western fast food. In women, marriage is associated with a higher risk of CKD.

**CONCLUSIONS:**

CKD is strongly associated with ageing, underlying diseases, smoking and drinking. Hypertension, elevated lipids, or diabetes are all risk factors that could be prevented or detected and treated. The Ministry of Public Health should encourage Thai people to consume healthy food, maintain a normal weight, stop smoking and drink alcohol in moderation, all of which will help prevent CKD.

## 1. Introduction

Chronic kidney disease (CKD) was already an important public health challenge around the globe when its leading cause was glomerulonephritis and interstitial nephritis (Barsoum, 2006). Now it is even more important because of the additional burden being added in developing countries as a consequence of emergent diabetes mellitus and hypertension (Haroun et al., 2003). Its incidence is increasing quickly worldwide due to the rapid increase in the prevalence of both diabetes (Wild, Roglic, Green, Sicree, & King, 2004) and hypertension (Kearney et al., 2005; Kearney, Whelton, Reynolds, Whelton, & He, 2004). A recent systematic review estimated the population prevalence of CKD ranges from 1.7% in China to 8.1% in the USA (McCullough et al., 2012). However, the estimates for developing countries are thought to be far below the actual rates and if so the demand for renal replacement services can be expected to rise dramatically over the next decade.

In Thailand, CKD is becoming a major public health burden since its incidence is alarmingly rising. When defined as a glomerular filtration rate (GFR) less than 60 ml per minute, the prevalence of CKD increased from 1.7% in 1985 to 6.8% in 1997 (Domrongkitchaiporn et al., 2005), 7.5% in 2007 (Satirapoj et al., 2013) and 8.6% in 2008 (Ingsathit et al., 2010). In addition, CKD is a major risk factor for cardiovascular disease (CVD) (de Zeeuw, Hillege, & de Jong, 2005; Mann, Gerstein, Pogue, Bosch, & Yusuf, 2001; Sarnak et al., 2003) and tends to progress to end-stage kidney disease (ESCKD) (Keith, Nichols, Gullion, Brown, & Smith, 2004) which demands high therapeutic costs and reduces life expectancy (Aekplakorn et al., 2008).

It has been known that CKD shares common traditional risk factors with CVD such as ageing, obesity, physical inactivity, hypertension, diabetes mellitus, dyslipidemia and smoking (Said & Hernandez, 2014). The risk of CKD increases as age increases (Tonelli & Riella, 2014) and females tend to have a higher prevalence than males (McCullough et al., 2012). Obesity (Garland, 2014; Satirapoj et al., 2013) and physical inactivity (S. Hallan et al., 2006; Stengel, Tarver-Carr, Powe, Eberhardt, & Brancati, 2003) increase risk to develop CKD. In addition, hypertension (Haroun et al., 2003; Major et al., 2014), diabetes (Metsarinne et al., 2014; Wang et al., 2014) and high lipids (Domrongkitchaiporn et al., 2005) are independent risk factors for CKD. Cigarette smoking (S. Hallan et al., 2006; Haroun et al., 2003) significantly increases risk of CKD while alcohol consumption (Stengel et al., 2003) is not associated with CKD. Even though the prevalence of smoking is tending to decline in Thailand, its intensity is increasing (Pachanee et al., 2011) so smoking remains an important risk factor for CKD in that setting.

The health-risk transition underway in Asia is associated with economic development and includes falling birth and death rates. The net result is an ageing population and increasing rates of non-communicable diseases (Shetty, 2012; Sleigh, Seubsman, & Bain, 2008). For example, in Thailand, the prevalence of overweight and obesity is rapidly rising. The third (2004) National Health Examination Survey (NHES) revealed that the prevalence of class I (25 ≤ BMI < 30) and class II (BMI ≥ 30) obesity in adults reached 18.2% and 7.5% respectively (Aekplakorn et al., 2007). Rapid socio-economic growth has urbanised many Thais with nearly half the population now living in urban areas with a tendency to sedentary lifestyles (Lim et al., 2009), increasing hypertension (Thawornchaisit et al., 2013b) and diabetes mellitus (Aekplakorn et al., 2011).

In Thailand, studies of chronic kidney disease are very limited and have been restricted to specific high risk groups (Domrongkitchaiporn et al., 2005; Satirapoj et al., 2013). Our large cohort study represents the Thai population across all geographical areas and measures a wide array of health risk factors for chronic kidney disease. Here we report on trends and associated CKD risks detected.

## 2. Methods

### 2.1 Data and Study Population

The Thai Cohort Study (TCS) is an on-going project investigating the health-risk transition as the country moves from a traditional pattern of infectious diseases to emerging chronic diseases. Started in 2005, the study recruited 87 143 distance-learning students enrolled at Sukhothai Thammathirat Open University (STOU). Details on study methodology and cohort characteristics have been described previously (Seubsman, Yiengprugsawan, Sleigh, & Thai Cohort Study, 2012; Sleigh et al., 2008). The study respondents well represented the STOU student body, and general Thai adult population, with similar median age, median income, country-wide residence and ethnic diversity (Sleigh et al., 2008). However, a higher proportion of cohort members resided in urban areas compared to national averages (51.8% vs. 31.1%) and they were also younger and better educated than most Thai adults. As such TCS members represented the Thais of the future, ahead of their compatriots on the health-risk transition path (Sleigh et al., 2008).

Data collected were based on a 20-page mail-out questionnaire that included a wide range of topics: demography, socio-economic status (SES), personal health (history of chronic kidney disease, diabetes mellitus, high blood lipids, high blood pressure, various cancers), goiter, epilepsy, asthma, arthritis, chronic bronchitis, and depression/anxiety. As well there were questions on hearing, vision or dental impairment and, usage of health services, social networks and personal well-being, health-related behaviour and family background.

### 2.2 Variables and Categories

Analyses were carried out with CKD as a dependent variable. Demographic factors (age, marital status, urbanization) and SES (income, household assets, and education), body mass index, underlying disease, and lifestyle factors (physical exercise, food preferences and intake, smoking and alcohol consumption) were independent variables.

Participants were grouped into three age categories: younger (≤29 years), middle (30-39 years) and older (≥40 years). Marital status was defined as married/living with a partner or single

Urbanization status was classified based on rural (R) or urban (U) residence when aged 10-12 years old and in 2005 thus producing 4 groups: lifelong ruralites (RR), urbanizers (RU), de-urbanizers (UR) and urbanites (UU).

Personal monthly income was divided into four categories: ≤7000 baht, 7001–10000 baht, 10001–20000 baht and >20000 baht. In 2005, one US dollar was equivalent to 42 baht so most of participants had quite low incomes. Household assets were classified into three categories: low (≤30,000 baht), medium (30,001-60,000) and high (>60,000).

Body mass index (BMI) was calculated and Asian cut-offs points for different body sizes were used, in accordance to guidelines of the International Obesity Task Force (Kanazawa et al., 2002). BMI classification was as follows: underweight (BMI < 18.50), normal (18.50 ≤ BMI < 23.00), overweight (23 ≤ BMI < 25.00), or obese (BMI ≥ 25).

Screen time (hours/day spent before TV or computer) and sitting time (hours/day spent sitting for any purpose) were assessed as proxies for sedentariness. Incidental exercise (frequency of housework or gardening), was categorised into 4 groups: ≤3 times per month; 1-2 times per week; 3-4 times per week; most days. Various forms of physical activity (at least 20 minutes of mild, moderate or strenuous exercise, 10 minutes or more walking sessions) were also included and recorded using 4-item ordinal categories ranging from never to ≥5 times per week.

We measured planned total physical activity based on the cohort members reporting the number of sessions per week of strenuous and moderate exercise for at least 20 minutes, and of walking for at least 10 minutes. We weighted the measures as follows: (2 × strenuous + 1 × moderate + 1 × walking) sessions per week. This weighting system is based on the recommendation of the International Physical Activity Questionnaire and the Active Australia Survey as used in other analyses of cohort data (Australian Institute of Health and Welfare, 2003; Banks, Lim, Seubsman, Bain, & Sleigh, 2011).

Smoking was self-reported and grouped into never, ex-smoker or current smoker. Alcohol consumption had four categories: never, ex-drinker, occasional drinker or current-drinker. Foods that could potentially influence CKD (deep fried, instant, roast or smoked, soybean products and soft drinks) were assessed for consumption frequency based on a five-point Likert scale ranging from less than once a month to once or more a day. Western-style fast food exposure was noted on a three-point scale from less than once to more than 3 times per month. Fruit and vegetable consumption were recorded as standard serves eaten per day.

### 2.3 Statistical Analyses

All analyses were performed using SPSS. The prevalence of CKD and its 95% confidence interval (CI) in the cohort was calculated for each category of all variables. To minimise demographic confounding we stratified analyses by sex. The association of CKD and each investigated health-risk factor was first analysed using a bivariate logistic regression. Variables that demonstrated significant association with CKD were then included in the multivariate logistic regression models. Statistical significance was set at p-value of <5%

## 3. Results

### 3.1 Study Population

The cohort had slight excess of females (54.7 vs. 45.3%) with the majority (60%) of participants in their 20s (Table 1). Central and North-eastern regions were better represented (24% and 21% respectively). About half of the TCS respondents were urban dwellers. Males had a better income but females had higher educational attainment. Household assets were distributed similarly for both groups with 40% of males and females belonging to the low assets group (≤30,000 baht), 30-31% in the medium asset group (30,001-60,000 baht), and 28-29% in the high assets group (>60,000 baht).

**Table 1:** Characteristics of the 87 143 participants in the Thai Cohort Study, 2005

Factor	Male		Female		Difference

	n	%	n	%	p-value^a^
**Demographic data**					
**Participants**	39485	45.3	47658	54.7	<0.0001
**Mean age-years (SD)**	32.24 (8.8)		29.07 (7.5)		<0.0001^b^
***Age group* (years)**					<0.0001
**≤29**	17721	44.9	28991	60.8	
**30-39**	13745	34.8	13571	28.5	
**≥40**	8015	20.3	5090	10.7	
***Marital status* (married/partnered)**					<0.0001
**No**	17941	46.8	27539	62.2	
**Yes**	20392	53.2	18713	37.8	
***Regions***					<0.0001
**Bangkok**	5715	14.6	9148	19.3	
**Central**	8867	22.7	12299	26	
**North**	7659	19.6	8095	17.1	
**North-east**	9554	24.4	8484	17.9	
**East**	2397	6.1	2930	6.2	
**South**	4933	12.6	6361	13.4	
***Urbanization status^c^***					<0.0001
**Rural–rural (RR)**	17607	45.3	20137	42.7	
**Rural–urban (RU)**	12619	32.4	14814	31.4	
**Urban–rural (UR)**	1699	4.4	2008	4.3	
**Urban–urban (UU)**	6978	17.9	10173	21.6	
**Socioeconomic status**					
***Education level***					<0.0001
**High school**	21750	55.1	20703	43.5	
**Diploma**	8903	22.6	14565	30.6	
**University**	8732	22.1	12253	25.8	
***Personal monthly income* (baht)^d^**					<0.0001
**≤7000**	13609	35.4	22026	47.4	
**7001–10000**	8821	22.9	10977	23.6	
**10001–20000**	10921	28.4	9648	20.8	
**>20000**	5152	13.4	3803	8.2	
***Household assets*^e^ (baht)**					<0.0001
**Low**	15911	40.5	19271	40.6	
**Medium**	12331	31.4	14271	30.1	
**High**	11024	28.1	13887	29.3	

a Chi-square test;

b unpaired *t* test;

c Location of residence (rural, R, or urban, U) at age 10-12 years and in 2005;

d At the time of the survey in 2005, US$1 = 42 Thai baht;

e Replacement value in Thai baht, divided into 3 groups: low ≤30 000, medium 30 001-60 000 and high >60 000.

### 3.2 Prevalence and Risk Factors of Chronic Kidney Disease

#### 3.2.1 Socio-Demographic Factors

The reported prevalence of CKD in the cohort in 2005 was 2.6% (2.5% of males and 2.7% of females) (Table 1). In unadjusted analyses, there were positive links between increasing age and increasing CKD prevalence in males but not in females. Married and partnered participants also had higher prevalence of CKD. There was no observed trend in urbanization status relative to CKD prevalence. However, after adjustment for other variables, older age emerged as a strong predictor for CKD in both sexes (OR range from 1 to 0.97 for male; 1-0.52 for females; p for both <0.01). Married or partnered women had 42% higher chances of getting CKD than single women but this association was not observed in men. Conversely, urbanite males (but not females) also had 40% lower odds of getting CKD compared to their ruralist counterpart.

There was no distinctive trend observed between socio-economic status and prevalence of CKD in bivariate analyses. After adjustment for other variables, this finding remains for females but not in males. The odds of having CKD in males decline as income and asset values increased (ORs drop from 1 to 0.71 for rising incomes, p<0.05; ORs from 1 to 0.78 as asset values increase, p<0.05).

#### 3.2.2 Lifestyle Factors

Lifestyle factors such as physical activities (housework and gardening), sedentariness (sitting time and television and computer watching time) and food consumption habits (deep fried food, instant food, soft drink, roasted or smoked food, fruit, vegetables and soybean products) were not associated with CKD (data not shown). The only food that was shown to be significantly associated with CKD was Western fast food, which if consumed ≥1 time/week could significantly elevate chance of getting CKD in men by 74% compared to <1 time/week (p trend <0.05).

Smoking and drinking were health related behaviours associated with CKD in both sexes. Previous and current smokers had from 11% to 85% higher odds of getting CKD. Similarly, previous and current drinkers also had increased odds ranging from 11% to 51% of getting CKD.

Two other lifestyle factors analysed were sleep duration and BMI classification; both were found to be associated with CKD among males but not females. Particularly, short sleep in males had 20% higher risk for CKD compared to normal sleeping of 7-8 hours (OR=1.21). The odds of getting CKD rose with increasing BMI: from underweight to normal and obese, odds ratios increased from 0.88 to 1.31 (p-trend <0.02).

#### 3.2.3 Health Conditions

The prevalence of CKD increased in both males and females who had diabetes, high blood lipids and hypertension. Participants with diabetes and hypertension had more than double the risk of CKD than those without (diabetes: OR=2.41 in males and 2.15 in females; hypertension: OR=2.3 in males and 2.95 in females). High lipids also increased the risk of CKD by 31% in females (p<0.05).

**Table 2:** Prevalence of chronic kidney disease and significant risk factors in male and female participants

	All males			All females		

	CKD(n)	%Prev^a^(95% CI)	AORs^b^ (95% CI)	CKD(n)	%Prev^a^(95% CI)	AORs^b^(95% CI)
Participants	1002	2.5 (2.3-2.7)		1283	2.7 (2.5-2.9)	
**Demography**						
*Age group*						
≤29 y	337	1.9 (1.7-2.1)	1	763	2.6 (2.4-2.8)	1
30-39 y	355	2.8 (2.6-3.0)	1.54 (1.27-1.86)	390	2.9 (2.7-3.1)	1.07 (0.92-1.25)
≥40 y	310	3.9 (3.7-4.1)	1.97 (1.55-2.5)	130	2.6 (2.2-3.0)	1.52 (1.18-1.97)
p-trend			<0.0001			<0.004
*Marital status* (married/partnered)						
No	395	2.2 (2.0-2.4)	1	649	2.4 (2.2-2.6)	1
Yes	572	2.8 (2.6-3.0)	1.02 (0.86-1.22)	588	3.1 (2.9-3.3)	1.42 (1.24-1.62)
*Urbanization status*						
Rural–rural (RR)	105	3.6 (3.0-4,2)	1	72	2.9 (2.3-3.5)	1
Rural–urban (RU)	135	2.8 (2.4-3.2)	0.86 (0.64-1.15)	144	2.9 (2.5-3.3)	1.0 (0.73-1.36)
Urban–rural (UR)	86	3.3 (2.5-4.1)	0.84 (0.61-1.17)	83	3.3 (2.5-4.1)	1.04 (0.73-1.47)
Urban–urban (UU)	101	2.1 (1.7-2.5)	0.59 (0.43-0.81)	145	2.7 (2.3-3.1)	0.86 (0.63-1.17)
p-trend			<0.007			0.54
**Socio-economic status**						
*Education level*						
High school	597	2.7 (2.5-2.9)	1	600	2.9 (2.7-3.1)	1
Diploma	197	2.2 (1.8-2.6)	0.87 (0.73-1.04)	373	2.6 (2.4-2.8)	0.89 (0.77-1.03)
University	206	2.4 (2.0-3.0)	0.87 (0.72-1.05)	305	2.5 (2.3-2.7)	0.95 (0.81-1.12)
p-trend			0.173			0.29
*Personal monthly income* (baht)^c^						
≤7000	340	2.5 (2.3-2.7)	1	600	2.7 (2.5-2.9)	1
7001-10000	194	2.2 (1.8-2.6)	0.81 (0.66-0.99)	310	2.8 (2.4-3.2)	1.05 (0.9-1.23)
10001-20000	296	2.7 (2.3-3.1)	0.81 (0.66-0.99)	242	2.5 (2.1-2.9)	0.89 (0.74-1.07)
>20000	145	2.8 (2.4-3.2)	0.71 (0.54-0.92)	106	2.8 (2.2-3.4)	0.96 (0.74-1.24)
p-trend			<0.049			0.40
*Household assets*^d^ (baht)						
Low	426	2.7 (2.5-2.9)	1	493	2.6 (2.4-2.8)	1
Medium	307	2.5 (2.3-2.7)	0.91 (0.76-1.08	380	2.7 (2.5-2.9)	1.02 (0.87-1.18)
High	259	2.3 (2.1-2.5)	0.78 (0.64-0.95)	403	2.9 (2.7-3.1)	1.09 (0.93-1.18)
p-trend			<0.04			0.57
**BMI classification^e^**						
Underweight (BMI <18.5)	66	2.8 (2.2-3.4)	0.88 (0.65-1.19)	283	2.8 (2.4-3.2)	1.15 (0.99-1.35)
Normal (18.5 ≤ BMI < 23)	473	2.5 (2.3-2.7)	1	708	2.5 (2.3-2.7)	1
Overweight (23 ≤ BMI < 25)	223	2.6 (2.2-3.0)	1.12 (0.93-1.35)	134	2.9 (2.3-3.5)	1.18 (0.97-1.45)
Obese (BMI ≥25)	230	2.6 (2.2-3.0)	1.31 (1.09-1.58)	143	3.0 (2.6-3.4)	1.06 (0.86-1.3)
p-trend			<0.02			0.17						

a Prevalence of chronic kidney disease;

b Adjusted Odd ratios from logistic regression models of chronic kidney disease adjusted for age, marital status, socioeconomic status, BMI, physical activities, underlying diseases, and personal behaviours;

c In 2005 US$1 = 42 Thai baht;

d Replacement value in Thai baht, divided into 3 groups: low ≤30 000, medium 30 001-60 000 and high >60 000;

e Asian standard BMI classification.

**Table 2:** (continued...)

	All males			All females		

	CKD(n)	%Prev^a^ (95% CI)	AORs^b^ (95% CI)	CKD(n)	%Prev^a^ (95%CI)	AORs^b^ (95% CI)
**Sedentary habits**						
*Sleeping duration*						
≤6 hr	405	2.9 (2.7-3.1)	1.21 (1.04-1.4)	401	2.6 (2.4-2.8)	0.95 (0.82-1.09)
7-8 hr	470	2.3 (2.1-2.5)	1	678	2.6 (2.4-2.8)	1
≥9 hr	127	2.7 (2.3-3.1)	1.11 (0.88-1.39)	204	3.1 (2.7-3.5)	1.18 (0.99-1.4)
p-trend			<0.048			0.073
**Physical activities**						
***Planned total physical activity***						
0-7 ses/w^c^	292	2.5 (2.3-2.7)	1	572	2.5 (2.3-2.7)	1
8-14 ses/w	285	2.5 (2.3-2.7)	0.99 (0.84-1.19)	364	2.7 (2.5-2.9)	1.07 (0.93-1.23)
≥15 ses/w	347	2.5 (2.3-2.7)	0.97 (0.82-1.16)	263	3.0 (2.6-3.4)	1.21 (1.03-1.41)
p-trend			0.94			0.065
**Underlying diseases**						
*Diabetes mellitus (type1 &2)*						
No	945	2.4 (2.2-2.6)	1	1261	2.7 (2.5-2.9)	1
Yes	57	8.9 (6.7-11.1)	2.41 (1.75-3.31)	22	8.5 (5.1-11.9)	2.15 (1.29-3.57)
*High lipids*						
No	815	2.3 (2.1-2.5)	1	1178	2.6 (2.4-2.8)	1
Yes	187	4.0 (2.8-5.2)	1.21 (0.99-1.48)	105	3.6 (3.0-4.2)	1.31 (1.03-1.65)
*Hypertension*						
No	839	2.3 (2.1-2.5)	1	1188	2.6 (2.4-2.8)	1
Yes	163	6.0 (5.0-7.0)	2.3 (1.88-2.8)	95	7.6 (6.2-9.0)	2.95 (2.31-3.76)
**Personal behaviours**						
*Smoking status*						
Never	378	2.1 (1.9-2.3)	1	1136	2.6 (2.4-2.8)	1
Ex-smoker	352	3.0 (2.6-3.4)	1.28 (1.09-1.5)	65	3.6 (2.8-4.4)	1.2 (0.91-1.59)
Cur-smoker^d^	208	2.6 (2.4-2.8)	1.11 (0.92-1.34)	22	4.7 (2.7-6.7)	1.85 (1.19-2.86)
p-trend			<0.012			<0.021
*Drinking status*						
Never	86	2.1 (1.7-2.5)	1	440	2.4 (2.2-2.6)	1
Ex-drinker	161	3.6 (3.0-4.2)	1.51 (1.12-2.03)	110	3.3 (2.7-3.9)	1.37 (1.08-1.72)
Occ – drinker^e^	641	2.4 (2.2-2.6)	1.12 (0.87-1.44)	701	2.8 (2.6-3.0)	1.2 (1.05-1.37)
Reg – drinker^f^	101	2.6 (2.0-3.2)	1.1 (0.79-1.53)	12	3.9 (1.7-6.1)	1.36 (0.7-2.64)
p-trend			<0.014			<0.014
**Food consumption habit**						
*Western fast food*						
<1 time/m	828	2.7 (2.6-3.4)	1	921	2.8 (2.6-3.0)	1
1-2 times/m	123	2.1 (2.3-2.7)	1.16 (0.94-1.44)	264	2.4 (2.2-2.6)	0.87 (0.77-1.04)
≥1 times/wk	18	1.3 (2.1-4.0)	1.74 (1.07-2.84)	79	3.2 (2.4-4.0)	1.18 (0.91-1.52)
p-trend			<0.026			0.12

a Prevalence of chronic kidney disease;

b Adjusted Odd ratios were from logistic regression models of chronic kidney disease adjusted for age, marital status, socioeconomic status, BMI classification, physical activities, underlying diseases, and personal behaviours;

c Sessions/week;

d Current-smoker;

e Occasional drinker;

f Regular drinker.

## 4. Discussion

We found chronic kidney disease associated with age, marital status, urbanisation, and income. CKD risk was higher among married women (but not men) and was lower for urbanised men (but not women). Increase in income or assets links to falling risk of CKD in males but not in females. Western fast food consumption, short sleep duration and obesity associated with rising CKD risk in males but not in females. Cigarette smoking and alcohol intake weakly associated with a higher risk of CKD in both sexes. Diabetes, high lipids, and high blood pressure linked to rising risk of CKD in both sexes. The overall prevalence of CKD (2.5% vs 8.9% vs 7.5%) among the cohort is much lower than that of other Thai studies (Ong-Ajyooth, Vareesangthip, Khonputsa, & Aekplakorn, 2009; Satirapoj et al., 2013) as TCS participants are younger.

The higher prevalence of CKD in females compared to males observed in this research was also previously found in studies in Thailand (Ingsathit et al., 2010; Ong-Ajyooth et al., 2009), Turkey (Suleymanlar et al., 2011), China (Chen et al., 2005), and America (Brown et al., 2003). The increased risk of CKD with ageing was also found in other studies in China (Zhang et al., 2012), in the US (Coresh, Astor, Greene, Eknoyan, & Levey, 2003; Coresh et al., 2005), and in Turkey (Suleymanlar et al., 2011). Effects of ageing on kidney diseases could have been due to decline in kidney function. Glomerular filtration rate (GFR) fell from 0.36 ml/min/1.73m^2^/year as estimated amongst the Japanese (Imai et al., 2008) to 1.03 ml/min/1.73m^2^/year among the Norwegians (Eriksen & Ingebretsen, 2006).

Socio-economic status (SES) has a negative effect on CKD as results showed in this study and in others conducted in India (Anupama & Uma, 2014), America (Klag et al., 1997; Krop et al., 1999; Perneger, Whelton, & Klag, 1995; White et al., 2008), Thailand (White et al., 2008) and Sweden (Fored et al., 2003). The effect of SES on CKD might have been due to poor dietary habits of lower SES groups. Crews et al. (2012) suggested that low income people may have poor diet and biological and psychological states which may lead to autonomic nervous system impairment reducing the ability to tolerate external stress which may cause endothelial injury. Our results showed that diet, particularly the consumption of Western food, is related to increased risk of CKD. Western fast foods contain high level of sugar, salt, fat and protein from red meat (Odermatt, 2011) as well as a high dietary acid load; all of which were demonstrated to increase the risk of kidney damaged through albuminuria (Levey et al., 1996; Lin, Hu, & Curhan, 2010; McMahon et al., 2013).

Obesity and short sleep are associated with an increased risk of CKD, especially in males. A large longitudinal study of 320,252 Americans showed that compared with participants who had normal weight, the odds to develop CKD for those who were overweight (25> BMI <30 kg/m^2^), or class I (30 ≥ BMI <35), II (35≥ BMI <40) or extreme (BMI ≥40) obesity were greater by 1.87, 3.57, 6.12 and 7.07 times, respectively (Hsu, McCulloch, Iribarren, Darbinian, & Go, 2006). A higher BMI category was associated with greater decline in kidney function (Grubbs et al., 2014). It is likely that obesity aggravates glomerular hyper-infiltration (Wuerzner et al., 2010) and chronic inflammation (Nolan, O’Meara, & Godson, 2013). Meanwhile, a retrospective cohort study of 6,834 Japanese employees showed that a self-reported short sleep duration (<5 hours per night) was associated with 28% increased risk of CKD (Yamamoto et al., 2012). Short sleep was also associated with hypertension (Gottlieb et al., 2006), overweight and obesity (Yiengprugsawan et al., 2012), which are the major risk factors for CKD as they lead to endothelial dysfunction and glomerular hypertension (Ohkuma et al., 2013).

Other health-related behaviours investigated in our study include cigarette smoking and alcohol drinking; both were strongly associated with a higher risk of CKD in both sexes. This is consistent with other reports including a cross-sectional study in the Netherlands (Pinto-Sietsma et al., 2000), a five-year prospective cohort study in Australia (White et al., 2009), community-based longitudinal studies in Norway (S. I. Hallan & Orth, 2011), and other studies in the USA (Fox et al., 2004; Haroun et al., 2003; Shankar, Klein, & Klein, 2006). Smoking leads to a higher risk of CKD through acute effects particularly an increased intra-glomerular blood pressure and chronic effects including endothelial cell dysfunction (Orth, 2004), proinflammation, oxidative stress, glomerulosclerosis and tubular atrophy (Mirrakhimov, 2012). Alcohol consumption may aggravate renal papillary necrosis, glomerulonephritis and rhabdomyolysis induced renal failure (White et al., 2009).

We also found a strong association between CKD and other health conditions including diabetes, hyperlipidaemia, and hypertension, in both males and females. The association between diabetes and CKD depends on the onset and duration of the diabetes. In other reports, individuals with juvenile diabetes (type I) had a 14.4% incidence of CKD after 10-year (Klein, Klein, Moss, Cruickshanks, & Brazy, 1999) while those with adult-onset diabetes (type II) had a 41% incidence after 20-year (Larson et al., 2000). It is also been reported that individuals with diabetes for more than five years had 4.65 times higher odds of developing nephropathy compared to those with less than one year duration (Goldschmid, Domin, Ziemer, Gallina, & Phillips, 1995). Diabetes leads to micro-albuminuria and without treatment it will progress to nephropathy after 15-20 years (Lea & Nicholas, 2002). A short term high blood sugar in diabetics also associated with a decreased glomerular filtration rate (GFR) with increased risk of CKD (Wang et al., 2014).

Other cohort studies in the US have demonstrated an association between hyperlipidaemia and CKD (Muntner, Coresh, Smith, Eckfeldt, & Klag, 2000; Schaeffner et al., 2003). Schaeffner et al. (2003) suggested that hyperlipidaemia might have a role in the pathogenesis of glomerulosclerosis and tubulointerstitial changes, while high serum cholesterol and triglyceride linked to lower GFR (Pechter et al., 2003).

A strong association between hypertension and CKD has also been shown in a cross-sectional study (Ingsathit et al., 2010) and in a small 12-year longitudinal study of electricity generator employees (Domrongkitchaiporn et al., 2005) in Thailand. In a Korean study, the risk of CKD development was 1.59 and 2.27 for pre-hypertension and hypertension respectively, compared to normotensive participants (Kim, Lim, & Park, 2012). Having both diabetes and hypertension appears to aggravate the risk of CKD even further. In African-Americans with diabetes, the risk of developing nephropathy was 2.64 times in participants with hypertension when compared to those without (Goldschmid et al., 1995). The rising incidence of diabetes (Aekplakorn et al., 2011) and hypertension (Thawornchaisit et al., 2013b) in Thailand as a result of an increased prevalence of obesity increases the risk of CKD.

### 4.1 Strengths and Limitations

Our study is based on a very large Thai cohort which evaluates most of the risk factors influencing CKD. We adjusted for confounding factors and expect risk factor associations identified are accurate. The participants represent the Thai population well in socio-demography, socio-economy and geography even though they are younger, more educated and have a better health (Seubsman et al., 2012; Sleigh et al., 2008). Data collecting by self-reported questionnaire from such an informative group is cost-effective and has high utility for a large national survey.

However, our analysed data were collected at one point in time so we cannot definitively establish causality. In addition, the data are based on self-report which may have recall or measurement error. However, the validity of self-reported weight and height in this population is actually high (Lim, Seubsman, & Sleigh, 2009) and the validity of self-reported hypertension is also acceptable (Thawornchaisit et al., 2013a). These validation studies imply self-report of health and disease by STOU participants is generally reliable for analysing in a large national cohort study.

In conclusion, in both males and females, CKD is strongly associated with ageing, cigarette smoking, drinking alcohol and having underlying diseases including diabetes mellitus, high lipids and hypertension. In males, ruralization, low income, low household asset, a higher BMI, short sleeping and having Western fast food are related to an increased CKD. Marriage increases the risk of CKD in females.

CKD is a major public health challenge in Thailand since diabetes, hypertension and obesity are rapidly rising and people are ageing. Some controls are needed and taxation could be used to tackle obesity, cigarette smoking and drinking alcohol. Healthy food consumption and maintenance of normal BMI will prevent Thai people suffering from chronic diseases, especially cardiovascular, CKD and diabetes.
